# Commentary: Successful treatment of refractory palmoplantar pustular psoriasis with apremilast: a case series

**DOI:** 10.3389/fmed.2023.1283313

**Published:** 2023-10-30

**Authors:** Madison Hackley, Amanda Loesch, Nicholas Brownstone, Sylvia Hsu

**Affiliations:** ^1^Lewis Katz School of Medicine, Temple University, Philadelphia, PA, United States; ^2^Temple University Health System, Inc., Philadelphia, PA, United States

**Keywords:** palmoplantar pustular psoriasis (PPP), apremilast, psoriasis, psoriatic arthritis, palmoplantar psoriasis

Recent reports highlight the success of apremilast in the treatment of palmoplantar pustular psoriasis (PPP). A case series published in *Frontiers in Dermatology* in October 2020 demonstrated that patients with refractory PPP had substantial improvement in PPP after administration of aprelimast (1). Soon after the publication of this article, a handful of other case series found similar data regarding the promising role of apremilast in the treatment of PPP. Additionally, in February 2023, a systematic review and meta-analysis demonstrated apremilast is an effective treatment option for PPP (2). At the time of this publication, there are ongoing clinical trials exploring the efficacy of apremilast in the treatment of PPP (3).

This retrospective case series published in Frontiers in Dermatology evaluated the response of six patients with PPP to apremilast treatment. Five of these patients also suffered from psoriatic arthritis (PsA). All participants had previously tried and failed treatment with topical steroids, PUVA, methotrexate, and at least four systemic anti-inflammatory medications. The patients were subsequently titrated to a maintenance dose of 60 mg of apremilast daily. The success of this medication on the treatment of PPP was evaluated using the physician global assessment (PGA). Four patients began the study with a PGA rating of 3 and the remaining two patients had a rating of 4.

The results were promising, indicating that all patients experienced an improvement in symptoms within the first month. After a year and a half, four of the patients improved to a PGA score of 1 and two patients stopped the medication due to adverse effects, most notably lack of improvement of psoriatic arthritis. Out of these patients, a few stopped the drug at some point for either personal or side effect reasons. After doing so, PPP recurred in all patients. If the drug was then restarted, the condition cleared rapidly.

A limitation of this study was its non-randomized and non-blinded nature. Additionally, the series involved only six patients, making the sample size small. Despite its limitations, the fact that the PPP recurred, when stopping the medication and significantly improved when restarting, demonstrates that apremilast may play a significant role in the treatment of PPP.

The majority of the patients in this study reported joint pain prior to starting apremilast and the study did not indicate that the drug improved psoriatic arthritis. In fact, one patient stopped the medication and switched to a different medication class due to severe joint pains. Patients with PPP have a significantly lower quality of life, and that is in part due to the concomitant joint pains, that is experienced by some of these patients. It may be true that apremilast helps to clear the cutaneous manifestations, however, leads to no improvement in the joint pains. The ideal treatment for PPP would equally address both the cutaneous and joint components of the disease process.

The biologic agent, Spevigo^Ⓡ^ (spesolimab), was recently FDA-approved for the treatment of generalized pustular psoriasis (GPP). Spesolimab is an anti-IL-36 receptor monoclonal antibody with a favorable safety profile that causes rapid skin pustular clearance in patients with GPP (4). Imsidolimab is another promising new treatment for GPP. Imsidolimab is also an anti-IL-36 receptor monoclonal antibody that has been shown to induce rapid and sustained improvement in GPP flares (5). It will be interesting to see how these newly approved biologic therapies used in the treatment of GPP work for patients with PPP.

Additionally, apremilast was not shown to be as effective in the treatment of plaque psoriasis as medications, such as deucravacitinib, a selective tyrosine kinase 2 inhibitor, in recent randomized control trials (6). A significantly higher percentage of patients treated with deucravacitinib showed >75% reduction from their baseline Psoriasis Area and Severity Index and Physician's Global Assessment score compared to those treated with apemilast (6). The less than favorable results of apremilast for the treatment of plaque psoriasis raises the question about apremilast's long-term success for PPP treatment. Despite apremilast's success in PPP treatment as reported in the case series, it is important to continue to explore potentially more efficacious treatment options for PPP.

Although apremilast represents a large step forward in the treatment of PPP, dermatologists must still exercise caution when utilizing this medication and pay careful attention to each patient's individual co-morbidities. Personalized treatment plans for patients with PPP are essential. Apremilast has the benefit of being categorized as a non-immunosuppressive drug, but it is possible there are more efficacious treatments than apremilast for PPP on the horizon. The authors are eager to see the results of apremilast for treatment of PPP in ongoing clinical trials and look forward to improved treatment options to offer our patients suffering from PPP.

## Author contributions

MH: Conceptualization, Writing—original draft, Writing—review and editing. AL: Conceptualization, Writing—original draft, Writing—review and editing. NB: Conceptualization, Supervision, Writing—review and editing. SH: Supervision, Writing—review and editing.
